# Phase separation of EML4–ALK in firing downstream signaling and promoting lung tumorigenesis

**DOI:** 10.1038/s41421-021-00270-5

**Published:** 2021-05-11

**Authors:** Zhen Qin, Honghua Sun, Meiting Yue, Xinwen Pan, Liang Chen, Xinhua Feng, Xiumin Yan, Xueliang Zhu, Hongbin Ji

**Affiliations:** 1 State Key Laboratory of Cell Biology, Shanghai Institute of Biochemistry and Cell Biology, Center for Excellence in Molecular Cell Science, Chinese Academy of Sciences, Shanghai, China; 2 University of Chinese Academy of Sciences, Beijing, China; 3 School of Life Science and Technology, Shanghai Tech University, Shanghai, China; 4 Institute of Life and Health Engineering, Jinan University, Guangzhou, Guangdong China; 5 Life Sciences Institute, Zhejiang University, Hangzhou, Zhejiang China; 6 School of Life Science, Hangzhou Institute for Advanced Study, University of Chinese Academy of Sciences, Hangzhou, Zhejiang China

**Keywords:** Non-small-cell lung cancer, Molecular biology

## Abstract

EML4–ALK fusion, observed in about 3%–7% of human lung adenocarcinoma, is one of the most important oncogenic drivers in initiating lung tumorigenesis. However, it still remains largely unknown about how EML4–ALK fusion exactly fires downstream signaling and drives lung cancer formation. We here find that EML4–ALK variant 1 (exon 1–13 of EML4 fused to exon 20–29 of ALK) forms condensates via phase separation in the cytoplasm of various human cancer cell lines. Using two genetically engineered mouse models (GEMMs), we find that EML4–ALK variant 1 can drive lung tumorigenesis and these murine tumors, as well as primary tumor-derived organoids, clearly show the condensates of EML4–ALK protein, further supporting the findings from in vitro study. Mutation of multiple aromatic residues in EML4 region significantly impairs the phase separation of EML4–ALK and dampens the activation of the downstream signaling pathways, especially the STAT3 phosphorylation. Importantly, it also significantly decreases cancer malignant transformation and tumor formation. These data together highlight an important role of phase separation in orchestrating EML4–ALK signaling and promoting tumorigenesis, which might provide new clues for the development of clinical therapeutic strategies in treating lung cancer patients with the EML4–ALK fusion.

## Introduction

Non-small cell lung cancer (NSCLC) is one of the most common cancers worldwide with high incidence and mortality^1^. According to the pathological classification, NSCLC can be categorized into three subtypes: adenocarcinoma (ADC), squamous cell carcinoma (SCC), and large cell carcinoma (LCC)^2^. NSCLC is frequently associated with oncogenic driver mutations which significantly contribute to tumorigenesis and cancer progression. For example, oncogenic mutations of epidermal growth factor receptor (EGFR), v-Ki-ras2 Kirsten rat sarcoma viral oncogene (KRAS), and anaplastic lymphoma kinase (ALK) fusions are the most frequent oncogenic drivers in NSCLC^3^.

The EML4–ALK fusion was initially discovered in Japanese NSCLC patients by Soda and colleagues in 2007^4^. Later study showed that about 3%–7% of NSCLC patients harbor EML4–ALK fusion^5^. ALK belongs to the receptor tyrosine kinase (RTK) family and the full length of ALK protein contains 1620 amino acids^6^. The ALK protein is comprised of three domains, including an extracellular domain (ECD), a transmembrane domain, and an intracellular domain (ICD)^7^. ALK expression is physiologically limited to embryonic stage, exclusively in the embryonic nervous systems, small intestine, and testis^8^. As a fusion partner of ALK, echinoderm microtubule-associated protein-like 4 (EML4) belongs to the echinoderm microtubule-associated protein-like family. EML4 comprises an N-terminal basic region, a hydrophobic echinoderm microtubule-associated protein-like protein (HELP) domain, and a tryptophan-aspartic acid (WD) repeat domain^9^. Previous study showed that the EML4 protein might be involved in the process of microtubule formation^9^. There are at least 15 EML4–ALK variants have been reported thus far and they uniformly contain the entire intracellular kinase domain of ALK which is encoded by exons 20–29^10^. Among all the EML4–ALK variants, EML4–ALK variant 1 is the most frequent form that accounts for about 43% patients^11^.

Unlike ALK as a membrane protein, the EML4–ALK fusion lacks the transmembrane domain and frequently localizes in the cytoplasm or microtubules^10^. By far two genetically engineered mouse models (GEMMs) of EML4–ALK have been established. Soda et al. generated the EML4–ALK mice, in which the EML4–ALK expression is driven by the surfactant protein C (SPC) promoter^12^. In another study, Pyo et al. developed a transgenic mouse model with the tamoxifen-inducible EML4–ALK expression^13^. The major downstream signaling pathways of EML4–ALK fusion include the mitogen-activated protein kinase (MAPK), the phosphoinositide-3-kinase (PI3K), and the signal transducer and activator of transcription 3 (STAT3) pathways^14^. Moreover, Zhang et al. recently found that ALK could inhibit TGF-β signaling through phosphorylating SMAD4 on tyrosine residue 95^15^. Activation of these signaling pathways could promote tumor cell survival, proliferation, and angiogenesis^16^.

Recent studies have highlighted the important role of protein phase separation in the formation of non-membranous organelles or compartments in both the nucleus and cytoplasm^17–20^. Phase separation is not only a simple physicochemical process but also regulates biological functions and activities^20,21^. However, whether protein phase separation could impact tumorigenesis, remains unclear.

We here demonstrate that EML4–ALK variant 1 forms condensates via phase separation in human cancer cell lines, murine lung tumors as well as tumor-derived organoids. Our data show that the phase separation of EML4–ALK is important for firing downstream signalings, especially the STAT3 phosphorylation, and promoting tumorigenesis.

## Results

### Phase separation of EML4–ALK variant 1 in human cancer cell lines

**Fig. 1:**
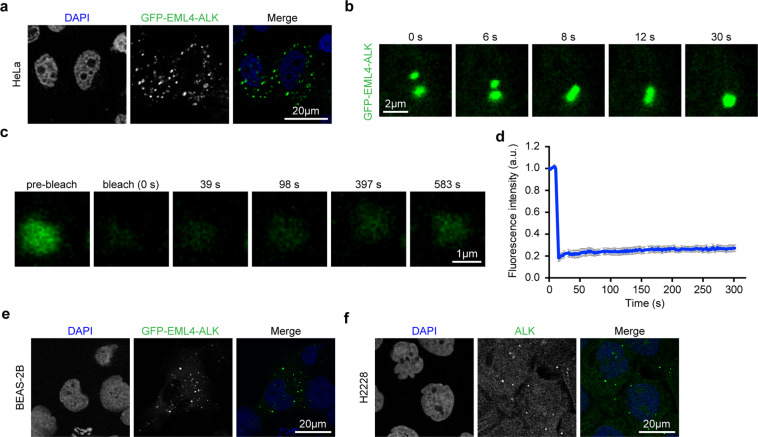
Phase separation of EML4–ALK variant 1 in human cancer cell lines. **a** HeLa cells were transfected with GFP–EML4–ALK for 24 h and the GFP–EML4–ALK was visualized by confocal microscopy. Nucleus was stained with DAPI (blue). Scale bar, 20 μm. **b** HeLa cells were transfected with GFP–EML4–ALK for 12 h and GFP fluorescence was monitored through live imaging. Snapshots at indicated time points showed the fusion event. Scale bar, 2 μm. **c** Representative FRAP images of GFP–EML4–ALK condensates in HeLa cells. The images were taken before and after photobleaching. Scale bar, 1 μm. **d** FRAP recovery curve of GFP–EML4–ALK condensates in HeLa cells. *n* = 12. Data were shown as mean ± SEM. **e** BEAS-2B cells were transfected with GFP–EML4–ALK for 24 h and the GFP–EML4–ALK was visualized by confocal microscopy. Nucleus was stained with DAPI (blue). Scale bar, 20 μm. **f** Immunofluorescence staining analysis of endogenous EML4–ALK in H2228 cells. ALK was indicated in green. Nucleus was stained with DAPI (blue). Scale bar, 20 μm.

To understand the localization and protein properties of EML4–ALK, we transiently expressed GFP–EML4–ALK variant 1 in HeLa cells, a commonly-used human cancer cell line. We clearly observed multiple near-spherical condensates positive for GFP–EML4–ALK in the cytoplasm (Fig. 1a). Intrigued by this finding, we next tested whether these condensates were formed through the liquid–liquid phase separation (LLPS). Through living cell imaging analyses, we found that the GFP–EML4–ALK condensates were able to undergo fusion, indicative of their liquid properties (Fig. 1b and Supplementary Movie S1). Fluorescence recovery after photobleaching (FRAP) assays revealed protein exchanges between the liquid droplets and the surroundings despite of a low exchange efficiency (Fig. 1c, d and Supplementary Fig. S1). As EML4–ALK is known as an important oncogenic driver in lung tumorigenesis, we overexpressed GFP–EML4–ALK variant 1 in BEAS-2B cells, a non-transformed human bronchial epithelial cell line and observed similar condensate formation (Fig. 1e). More importantly, when H2228 cells, an EML4–ALK fusion-containing lung cancer cell line, were immunostained with an anti-ALK antibody, we observed similar spherical condensates (Fig. 1f), suggesting that endogenous EML4–ALK also undergoes LLPS. We also performed the FRAP assays in BEAS-2B and H2228 cells and observed similar low protein exchange efficiency (Supplementary Fig. S2a, b). These data together suggest that EML4–ALK has an intrinsic capacity to phase separate into liquid-like condensates in various human cancer cell lines.

### Phase separation of EML4–ALK in murine tumors and organoids

**Fig. 2:**
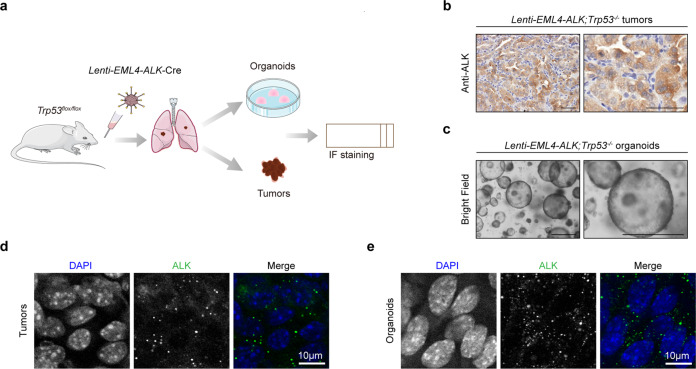
EML4–ALK forms condensates in lung tumors and tumor-derived organoids in *Lenti-EML4-ALK;Trp53*^−*/*−^ mouse model. **a** Schematic illustration of *Lenti-EML4-ALK;Trp53*^*−/*−^ mouse model. *Trp53*^*flox/flox*^ mice at 6–8 weeks were treated with 1 × 10^6^ PFU of *Lenti-EML4-ALK-Cre* lentivirus via nasal inhalation and analyzed 28 weeks afterward for immunofluorescence staining of lung tumors and tumor-derived organoids. **b** Representative photos for ALK immunostaining in *Lenti-EML4-ALK;Trp53*^−*/−*^ lung tumors. Scale bar, 50 μm. **c** Representative photos for *Lenti-EML4-ALK;Trp53*^*−/−*^ organoids derived from lung tumors. Scale bar, 500 μm. **d** Immunofluorescence staining analysis of EML4–ALK in *Lenti-EML4-ALK;Trp53*^−*/−*^ tumors. ALK was indicated in green. Nucleus was stained with DAPI (blue). Scale bar, 10 μm. **e** Immunofluorescence staining analysis of EML4–ALK in *Lenti-EML4-ALK;Trp53*^*−/−*^ organoids. ALK was indicated in green. Nucleus was stained with DAPI (blue). Scale bar, 10 μm.

**Fig. 3:**
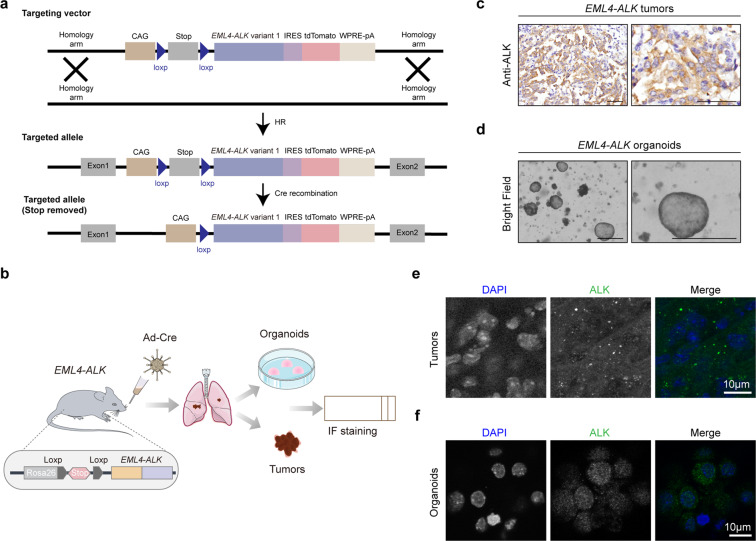
EML4–ALK forms condensates in *EML4–ALK* tumors and organoids. **a** Schematic illustration of the Rosa26-Loxp-Stop-Loxp-*EML4–ALK* mice. See “Materials and methods” for details. **b** Schematic illustration of *EML4–ALK* mouse model. *EML4–ALK* mice at 6–8 weeks were treated with 2 × 10^6^ PFU of Ad-Cre via nasal inhalation and analyzed 5 weeks afterward for immunofluorescence staining of tumors and tumor-derived organoids. **c** Representative photos for ALK immunostaining in *EML4–ALK* tumors. Scale bar, 50 μm. **d** Representative photos for *EML4–ALK* organoids derived from lung tumors. Scale bar, 500 μm. **e** Immunofluorescence staining analysis of EML4–ALK in *EML4–ALK* tumors. ALK was indicated in green. Nucleus was stained with DAPI (blue). Scale bar, 10 μm. **f** Immunofluorescence staining analysis of EML4–ALK in *EML4–ALK* organoids. ALK was indicated in green. Nucleus was stained with DAPI (blue). Scale bar, 10 μm.

To test whether the phase separation of EML4–ALK fusion exists in vivo, we took advantage of two GEMMs. In the first mouse model, we delivered the lentivirus carrying EML4–ALK variant 1 together with Cre into the *Trp53*^*flox/flox*^ mice through nasal inhalation as previously described^22^ (Fig. 2a). After 28 weeks of viral treatment (1 × 10^6^ PFU), these mice were sacrificed for pathological analysis and 3D tumor organoids culture (Fig. 2a, c). These tumors displayed high expression of EML4–ALK (Fig. 2b). Importantly, the EML4–ALK condensates were clearly detectable in these murine lung tumors (Fig. 2d) and tumor-derived organoids (Fig. 2e). In the second mouse model, we integrated the loxp-stop-loxp-EML4–ALK variant 1 transgene into the Rosa26 locus of C57BL/6 mice (Fig. 3a). After 5 weeks of nasal inhalation with 2 × 10^6^ PFU Ad-Cre, the mice were sacrificed for further analyses (Fig. 3b, d). Consistently, we found strong EML4–ALK expression in tumor areas (Fig. 3c). Obvious condensate formation of EML4–ALK proteins was observed in both murine lung tumors (Fig. 3e) and tumor-derived organoids (Fig. 3f). These data together provide in vivo evidence in supporting the phase separation of EML4–ALK.

### Phase separation of EML4–ALK depends on the EML4 region

**Fig. 4:**
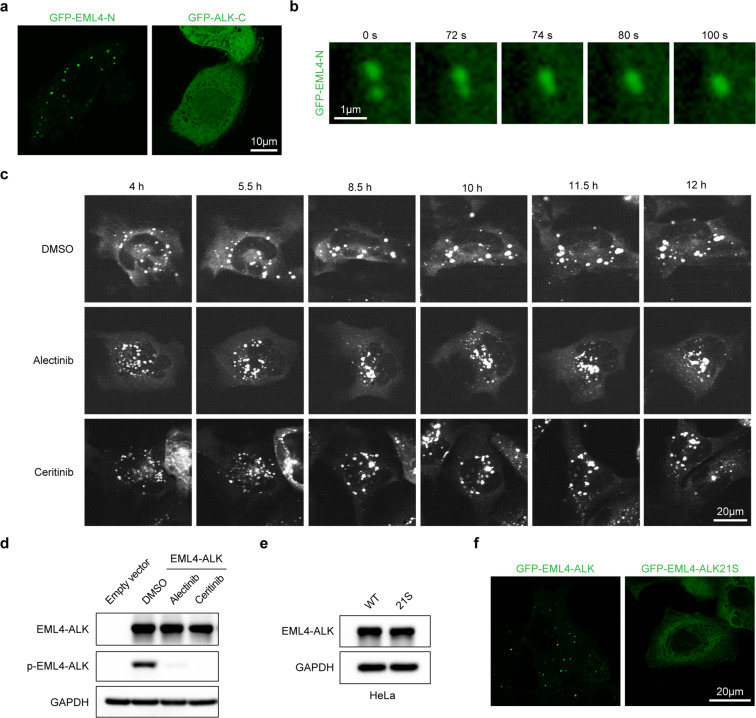
The EML4–ALK21S mutant fails to phase separate. **a** Representative fluorescent images of HeLa cells expressing GFP–EML4-N or GFP–ALK-C. Either GFP–EML4-N or GFP–ALK-C was visualized by confocal microscopy. Scale bar, 10 μm. **b** HeLa cells were transfected with GFP–EML4-N for 12 h and GFP fluorescence was monitored through live imaging. Snapshots at indicated time points showed the fusion event. Scale bar, 1 μm. **c** BEAS-2B cells were transfected with GFP–EML4–ALK for 24 h. Cells were treated with DMSO or ALK inhibitors, alectinib (500 nM), ceritinib (500 nM), and GFP fluorescence was monitored through live imaging for up to 12 h. Scale bar, 20 μm. **d** Western blot analysis of EML4–ALK phosphorylation after ALK inhibitor treatment for 12 h. **e** HeLa cells were transfected with GFP–EML4–ALK or GFP–EML4–ALK21S and analyzed by western blot. WT, GFP–EML4–ALK; 21S, GFP–EML4–ALK21S. **f** HeLa cells were transfected with GFP–EML4–ALK or GFP–EML4–ALK21S and analyzed by confocal microscopy. Scale bar, 20 μm.

We then asked which fusion partner contributed to the phase separation of EML4–ALK. We created two truncation constructs and found that only GFP–EML4-N was able to form condensates similar to GFP–EML4–ALK, whereas GFP–ALK-C showed a dispersed location in the cytoplasm (Fig. 4a). Through living cell imaging analyses, we found that the condensates of GFP–EML4-N also underwent fusion (Fig. 4b and Supplementary Movie S2). These findings indicate that EML4 region alone is sufficient for condensate formation.

EML4–ALK fusion leads to the constitutive activation of ALK kinase and results in the activation of downstream signaling pathways^23^. To further explore whether ALK kinase activity is necessary for condensate formation, we examined the dynamics of EML4–ALK condensates after ALK inhibitor treatment. Live imaging showed that either alectinib or ceritinib treatment had no significant impact upon the formation of EML4–ALK condensates (Fig. 4c, d). These data suggest that EML4–ALK condensate formation is potentially independent of its kinase activity.

Previous studies indicate that aromatic residues play an important role in promoting the phase separation of intrinsically disordered proteins^24,25^. We then generated the GFP–EML4–ALK21S mutant, in which most of the aromatic residues (9 tyrosine residues and 12 phenylalanine residues) were replaced in the EML4 region with serine residues (Supplementary Fig. S3). Western blot analysis showed comparable protein levels of GFP–EML4–ALK and GFP–EML4–ALK21S (Fig. 4e). In contrast to GFP–EML4–ALK, the GFP–EML4–ALK21S became dispersed in the cytoplasm when overexpressed in HeLa cells (Fig. 4f). These data support that the phase separation of EML4–ALK is dependent on the EML4 region.

### Phase separation is required for the EML4–ALK-induced hyperactivation of downstream signaling pathways

**Fig. 5:**
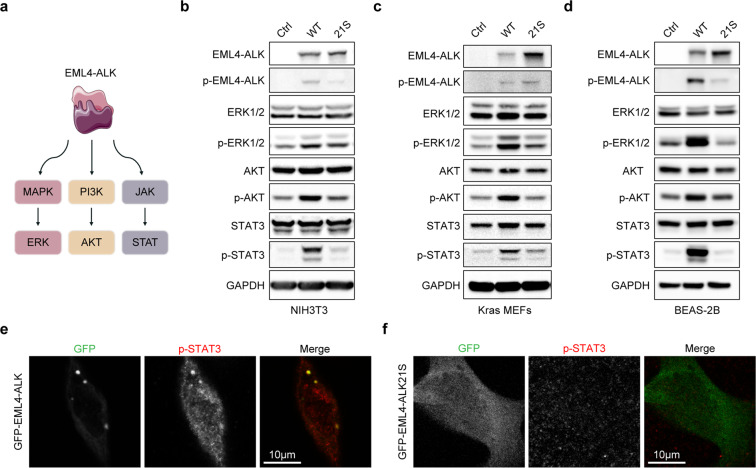
The 21S mutations markedly attenuate the EML4–ALK-induced hyperactivation of downstream signaling pathways. **a** Schematic illustration of EML4–ALK downstream signaling pathways. **b**–**d** Western blot analysis of AKT, ERK1/2, STAT3 phosphorylation. NIH3T3 (**b**), Kras MEFs (**c**) and BEAS-2B (**d**) cells, stably expressing EML4–ALK or EML4–ALK21S, were deprived of serum and glucose for 2 h and then subjected to western blot analysis. Ctrl, control; WT, GFP–EML4–ALK; 21 S, GFP–EML4–ALK21S. **e**, **f** H2228 cells were transfected with the GFP–EML4–ALK (**e**) or GFP–EML4–ALK21S (**f**) and analyzed by confocal microscopy. P-STAT3 was indicated in red. Scale bar, 10 μm.

As the phosphorylation of AKT, ERK1/2, and STAT3 are mainly mediated by three downstream signaling pathways of EML4–ALK^16^, we investigated whether the disruption of EML4–ALK condensate formation could affect the activation of EML4–ALK downstream signalings (Fig. 5a). Consistent with the previous report^23^, multiple cell lines stably expressing GFP–EML4–ALK displayed prominently elevated phosphorylation levels of AKT, ERK1/2, and STAT3 as compared to their parental control cells (Fig. 5b–d). In sharp contrast, the STAT3 phosphorylation levels were comparable between cells stably expressing GFP–EML4–ALK21S and the control cells (Fig. 5b–d). The phosphorylation levels of ERK1/2 and AKT in the stable cell lines of constitutively expressing GFP–EML4–ALK21S were also comparable to or only slightly exceeded those of control cells (Fig. 5b–d). These results indicate that the phase separation property of EML4–ALK is essential for its ability to hyperactivate these downstream pathways, especially STAT3 phosphorylation.

To further clarify the link between the STAT3 phosphorylation and the condensate formation, we overexpressed GFP–EML4–ALK and GFP–EML4–ALK21S in H2228 cells and performed immunostaining analysis. The overexpression of GFP–EML4–ALK led to the formation of condensates and the enrichment of p-STAT3 (Fig. 5e), whereas the overexpression of GFP–EML4–ALK21S failed to form condensates (Fig. 5f). This suggests a possibility that the EML4–ALK condensates hyperactivate the STAT3 signaling pathway through the direct recruitment of downstream component.

### Phase separation of EML4–ALK is critical for its oncogenic property

**Fig. 6:**
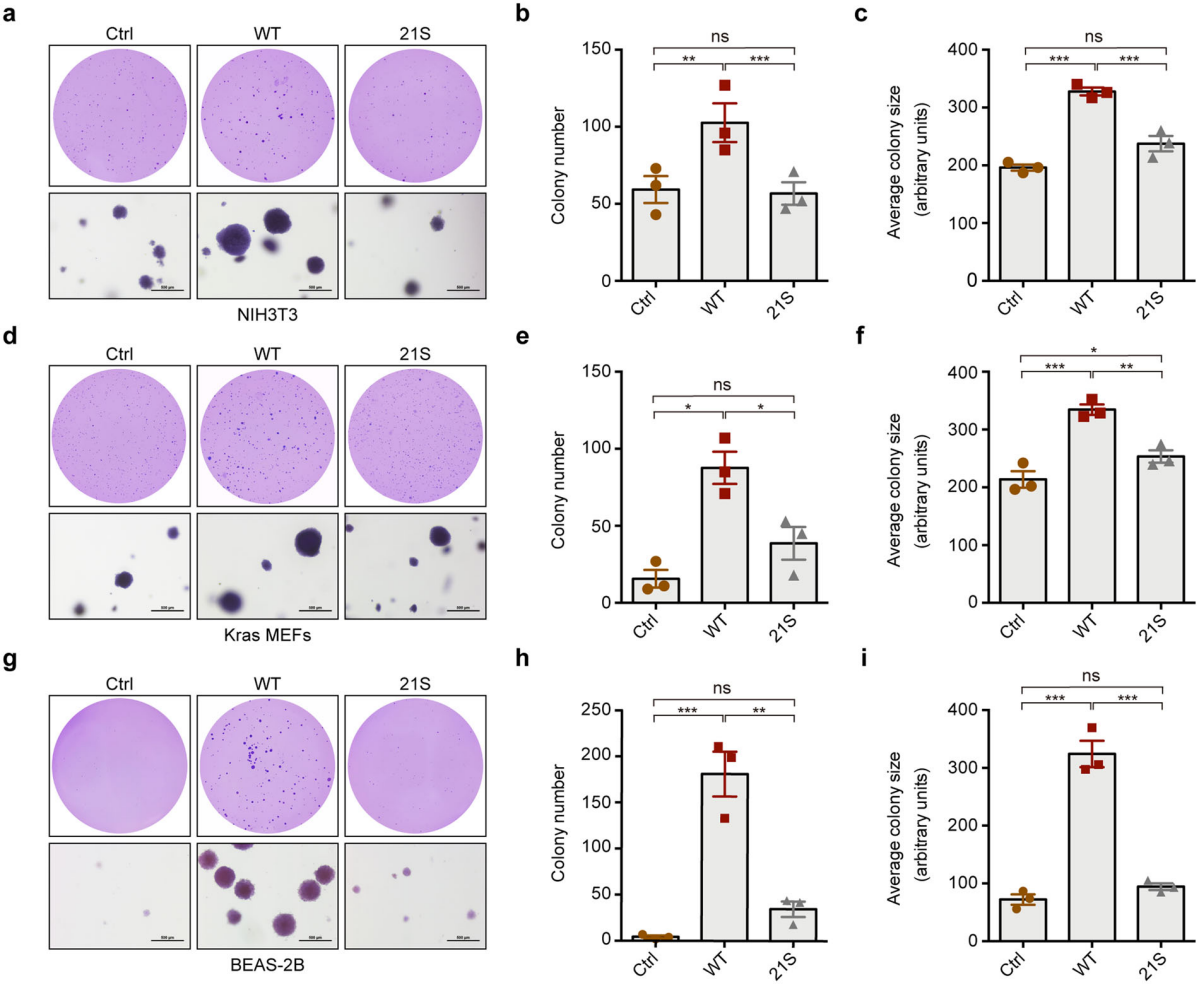
The 21S mutations impair the tumorigenic capability of EML4–ALK in soft-agar colony formation. **a**–**c** NIH3T3 cells were stably transfected with empty vector, EML4–ALK or EML4–ALK21S and analyzed by soft-agar colony formation assay. Representative images for soft-agar colonies (**a**). Scale bar, 500 μm. Statistical analysis of colony numbers (**b**). Statistical analysis of colony sizes (**c**). **d**–**f** Kras MEFs cells were stably transfected with empty vector, EML4–ALK or EML4–ALK21S and analyzed by soft-agar colony formation assay. Representative images for soft-agar colonies (**d**). Scale bar, 500 μm. Statistical analysis of colony numbers (**e**). Statistical analysis of colony sizes (**f**). **g**–**i** BEAS-2B cells were stably transfected with empty vector, EML4–ALK or EML4–ALK21S and analyzed by soft-agar colony formation assay. Representative images for soft-agar colonies (**g**). Scale bar, 500 μm. Statistical analysis of colony numbers (**h**). Statistical analysis of colony sizes (**i**). All data were shown as mean ± SEM. *P < 0.05; **P < 0.01; ***P < 0.001; ns: not significant. Ctrl, control; WT, GFP–EML4–ALK; 21S, GFP–EML4–ALK21S.

We next functionally characterized whether the disruption of EML4–ALK condensate formation disturbed the neoplastic transformation. In contrast to wild-type EML4–ALK, the EML4–ALK21S mutant displayed dramatically decreased capability in promoting soft-agar colony formation in NIH3T3 cells (Fig. 6a-c). Using Kras mouse embryonic fibroblasts (MEFs) and BEAS-2B cells, we observed similar decrease of transformation capabilities upon the overexpression of EML4–ALK21S mutant (Fig. 6d-i).

**Fig. 7:**
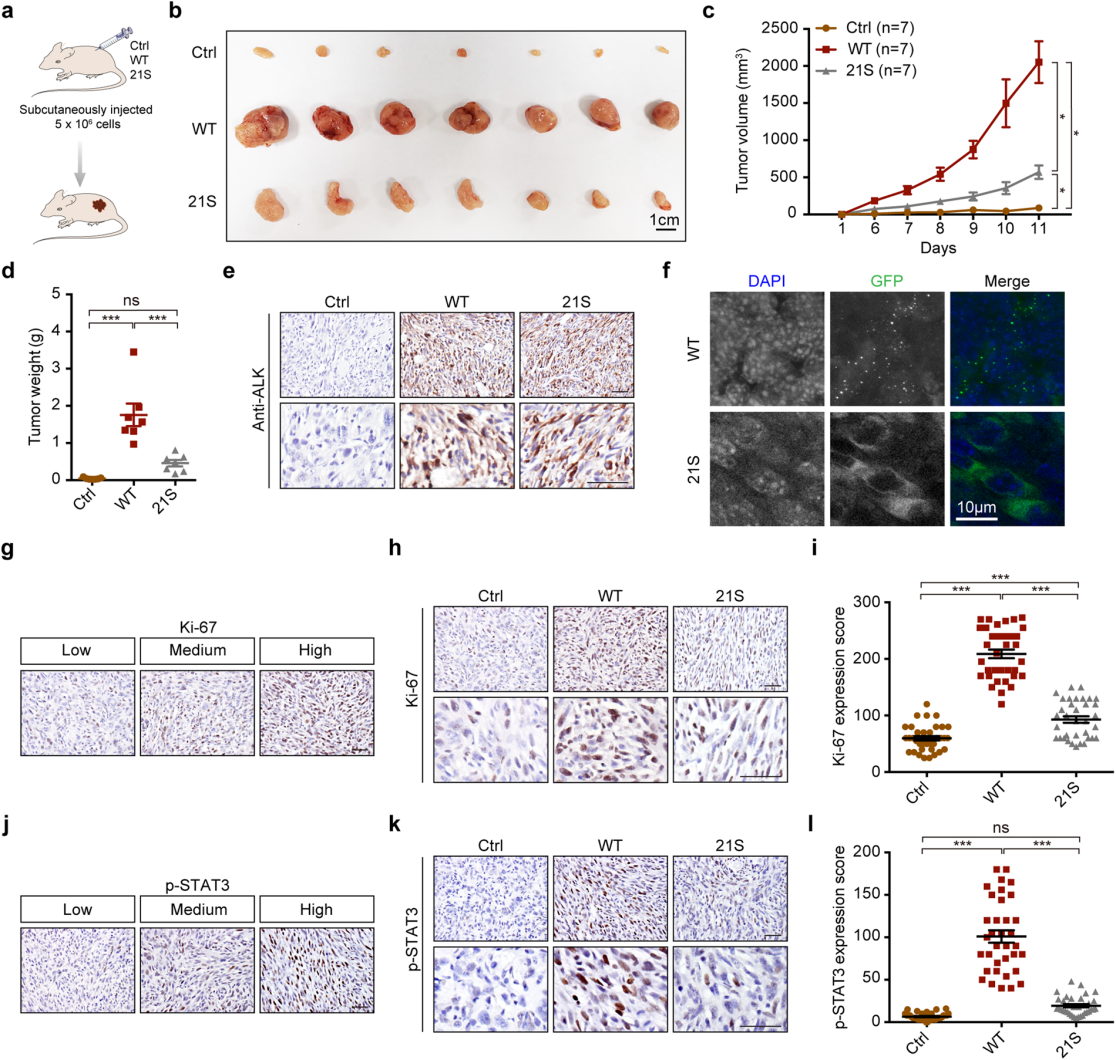
The EML4–ALK21S mutant exhibits impaired capability in tumor formation. **a** Schematic illustration of tumor formation assay in nude mice. See “Materials and methods” for details. **b** Photos of subcutaneous tumors derived from control, GFP–EML4–ALK and GFP–EML4–ALK21S groups. Scale bar, 1 cm. *n* = 7 for each group. **c** Growth curves of the subcutaneous tumors. **d** Statistical analysis of tumor weights. **e** Representative photos for ALK immunostaining in subcutaneous tumors derived from control, GFP–EML4–ALK and GFP–EML4–ALK21S groups. Scale bar, 50 μm. **f** Representative fluorescence photos for subcutaneous tumors derived from GFP–EML4–ALK and GFP–EML4–ALK21S groups. The GFP–EML4–ALK or GFP–EML4–ALK21S was visualized by confocal microscopy. Nucleus was stained with DAPI (blue). Scale bar, 10 μm. **g** Representative images of low, medium, high expression of Ki-67. Scale bar, 50 μm. **h** Representative photos for Ki-67 immunostaining in subcutaneous tumors derived from control, EML4–ALK and EML4–ALK21S groups. Scale bar, 50 μm. **i** Statistical analysis of Ki-67 staining. **j** Representative images of low, medium, high expression of p-STAT3. Scale bar, 50 μm. **k** Representative photos for p-STAT3 immunostaining in subcutaneous tumors derived from control, GFP–EML4–ALK and GFP–EML4–ALK21S groups. Scale bar, 50 μm. **l** Statistical analysis of p-STAT3 immunostaining. All data were shown as mean ± SEM. *P < 0.05; **P < 0.01; ***P < 0.001. Ctrl, control; WT, GFP–EML4–ALK; 21S, GFP–EML4–ALK21S.

We further performed in vivo tumor formation assay in nude mice using NIH3T3 cells with overexpression of wild-type EML4–ALK or the EML4–ALK21S mutant (Fig. 7a). The wild-type EML4–ALK was able to drive fast tumor growth even after 11 days of transplantation (Fig. 7c). In contrast, the EML4–ALK21S mutant group showed a significantly impaired tumor growth and dramatically decreased tumor sizes and weights (Fig. 7b–d), despite of comparable ALK expression (Fig. 7e). The EML4–ALK condensates were clearly present in wild-type EML4–ALK tumors but almost undetectable in EML4–ALK21S tumors (Fig. 7f). Consistently, proliferative cells indicated by Ki-67 positive staining were significantly decreased in EML4–ALK21S tumors (Fig. 7g-i). To further check the changes of downstream signalings, we conducted the immunostaining of p-AKT, p-ERK1/2, and p-STAT3. Compared to wild-type EML4–ALK tumors, the EML4–ALK21S tumors showed markedly reduced p-STAT3 levels (Fig. 7j–l). The p-ERK1/2 levels were also reduced but not as striking (Supplementary Fig. S4a–c), whereas the levels of p-AKT were barely detectable in all the tumors (Supplementary Fig. S4d). These data together demonstrate that EML4–ALK phase separation is required for the downstream STAT3 activation and neoplastic transformation.

## Discussion

Emerging evidence begins to link cancer-related genes to condensate assembly, indicative of the important role of phase separation in tumorigenesis^26^. Boulay et al. find that the phase separation of EWS–FLI1 fusion promotes the formation of super-enhancers and oncogenic transcriptional programs in Ewing sarcoma cancer^27^. Another study links phase separation to tumor suppressor. Bouchard et al. find that mutations in the tumor suppressor *SPOP* disrupt phase separation and lead to a buildup of cancer-promoting proteins^28^. EML4–ALK fusion is the most dominant fusion in lung cancer. Previous studies report that kinase activities of EML4–ALK are mainly dependent on dimerization or autophosphorylation of the kinase domain^29^. Thus, it remains largely unknown whether phase separation contributes to the oncogenic activation of EML4–ALK.

Our study here shows that EML4–ALK forms condensates via phase separation in human cancer cell lines and mouse lung tumors. We further find that phase separation plays an important role in EML4–ALK-driven tumorigenesis whereas the disruption of phase separation significantly impairs downstream signaling and neoplastic transformation. Fascinatingly, two recent studies show that EML4–ALK variant 1 or variant 3 could form cytoplasmic protein granules to regulate downstream pathways^30,31^. Tulpule et al. find low exchange of EML4–ALK variant 1 between the granules and the surroundings in FRAP assays^31^, which is consistent with our findings. Sampson et al. find that alectinib treatment promotes phase separation of EML4–ALK variant 3^30^. In contrast, we find that alectinib or ceritinib treatment shows no impact upon the phase separation of EML4–ALK variant 1. We reason the effect of alectinib in phase separation might work in the context of different forms of EML4–ALK fusions. Future study will be interesting to elucidate the detailed relationship between EML4–ALK kinase activity and phase separation. Moreover, Tulpule et al. demonstrate that EML4–ALK granules mainly regulate downstream MAPK pathway^31^. Interestingly, we find although all the three pathways are affected by the disruption of phage separation, the decrease of STAT3 phosphorylation seems most dramatic in multiple cell lines and tumors. It remains very interesting to investigate how the phase separation finely tunes the various downstream signaling of EML4–ALK in the future.

Collectively, our study demonstrates that phase separation is an important cellular process for EML4–ALK proteins and mediates the activation of downstream signaling pathways. Disruption of phase separation preferentially impairs the STAT3 phosphorylation and decreases the capability of malignant transformation. Our findings may provide a new approach for treating the EML4–ALK-positive lung cancer that aims to disrupt protein condensates.

## Materials and methods

### Mouse model

The *Trp53*^*flox/flox*^ mice were originally provided by Dr. Tyler Jacks (Cambridge, MA). The transgenic EML4–ALK mouse model was generated by CRISPR/Cas9 technology. We inserted CAG-EML4-ALK-IRES-tdTomato expression box at the site of the Rosa26 gene through homologous recombination. The brief process is as follows: Cas9 mRNA and gRNA were obtained by in vitro transcription, the homologous recombination vector (donor vector) was constructed by the In-Fusion cloning method, which contained a 3.3 kb 5′ homology arm, CAG-EML4-ALK-IRES-tdTomato, and 3.3 kb 3′ homology arm. Cas9 mRNA, gRNA, and donor vector were microinjected into the fertilized eggs of C57BL/6J mice to obtain F0 generation mice. All mice were kept in specific pathogen-free environment of Shanghai Institute of Biochemistry and Cell Biology, received humane care and treated in strict accordance with protocols approved by the Institutional Animal Care and Use Committee of the Shanghai Institutes for Biological Sciences, Chinese Academy of Sciences. Mice were treated with Ad-Cre virus at 2 × 10^6^ PFU or *Lenti-EML4-ALK-Cre* virus at 1 × 10^6^ PFU by nasal inhalation at 6–8 weeks of age.

### Plasmid construction

Full-length EML4–ALK was amplified and inserted into GFP-3x linker vector plasmid. Domain truncation constructs were generated by standard PCR-based cloning. Mutant plasmid was synthesized by Gene synthesis technology in shanghai Generay biotech Co., Ltd. All the constructs were verified by sequencing.

### Immunofluorescence and Fluorescent microscopy

Cultured cells or organoids were fixed with 4% paraformaldehyde in PBS for 15 min at room temperature. Fixed cells were permeabilized with 0.5% Triton X-100 in PBS for 15 min and blocked with 4% bovine serum albumin in TBST for 1 h. The cells or organoids were incubated with following antibodies: ALK (CST, 3633 S, 1:250), p-STAT3 (CST, 7145, 1:100) and washed three times with 4% bovine serum albumin. After incubation with secondary antibodies at room temperature for 1 h, the cells were washed three times with TBST. Then, the coverslips were mounted onto glass slides using fluorescent mounting medium. Confocal images were captured using a Leica TCS SP8 system with a HC PL APO CS2 63×/1.40 oil objective.

### Live-cell imaging

For live-cell imaging, cells were seeded in 35-mm glass-bottom dishes (D35-20-1.5-N, Cellvis). For imaging the droplet fusion, HeLa cells were transfected with plasmids for 12 h. Images were captured at 2 s intervals with a Zeiss LSM880 Airyscan microscope equipped with a 63× oil immersion objective. For live-cell imaging after ALK inhibitor treatment, BEAS-2B cells were transfected with plasmids for 24 h and treated with alectinib (MCE, 500 nM) and ceritinib (MCE, 500 nM). Continuous images were captured for 12 h.

### Fluorescence recovery after photobleaching (FRAP)

FRAP experiments in cells were carried out with following settings: region of interest (ROI) were bleached using a 405 nm diode, pre-bleach and post-bleach images were acquired with a 488 nm laser. Fluorescence recovery of GFP–EML4–ALK was monitored for 10 or 15 min with a time resolution of 2 s. Images were captured at 2 s intervals with a Zeiss LSM880 Airyscan microscope equipped with a 63× oil immersion objective.

### In vitro organoid culture

The mouse tumor organoids were established using previously described culture methods^32^. Briefly, mouse tumors were minced with scissors and digested in 1 mL of 5 mg/mL collagenase type II (Invitrogen) in Advanced DMEM/F12 (Gibco) and digested for 1–2 h at 37 °C with shaking. Dissociated cells were washed and then seeded in growth factor-reduced Matrigel (BD biosciences). Organoids were passaged at a 1:3 dilution every 4 days via trituration with glass Pasteur pipettes.

### Lentivirus production and infection

The production of lentivirus supernatant was described previously^33^. The cell lines NIH3T3 (ATCC) and Kras MEFs were maintained in DMEM (Hyclone) supplemented with 8% FBS, the cell lines BEAS-2B (ATCC) were maintained in RPMI-1640 supplemented with 8% FBS. For stable overexpression of EML4–ALK variant 1, the cells infected with virus were persistently maintained in medium with puromycin (2 µg/mL, Sigma).

### Western blot

Whole-cell lysates of cell lines were prepared in lysis buffer (10% SDS, 1 mM DTT, and glycerin) and incubated at 100 °C for 10 min. Equal volumes of proteins were resolved by SDS-PAGE and transferred onto PVDF membranes. Protein samples were probed with specific antibodies against ALK (CST, 3633 S, 1:2000), p-ALK (CST, 3341S, 1:1000), ERK (CST, 9102, 1:1000), p-ERK (CST, 4370, 1:1000), AKT (CST, 9272, 1:1000), p-AKT (CST, 4070, 1:1000), STAT3 (CST, 9139, 1:1000), p-STAT3 (CST, 7145, 1:1000), or GAPDH (Abclonal, AC002, 1:5000). Protein expression was assessed by Pierce ECL Western Blotting Substrate (Thermo Fisher Scientific) and detected on SAGECREATION (Sage Creation Science Co, Beijing).

### Immunostaining

Immunostaining was performed as previously described^34^. Paraffin-embedded tissues were incubated with following antibodies: ALK (CST, 3633 S, 1:250), Ki-67 (Novus, NB500–170, 1:1000), p-ERK (CST, 4370, 1:400), p-AKT (CST, 4070, 1:400), p-STAT3 (CST, 7145, 1:400). The IHC expression score was evaluated by counting Ki-67-positive, p-STAT3-positive and p-ERK1/2-positive staining at high-power field (HPF) for 35 fields for each group.

### Soft agar colony formation assay

For soft agar assay, a bottom layer of 1% agar with complete medium is solidified first, followed by an upper layer containing 5000 cells suspended in 0.4% medium-agar mixture in 6-well plates. After 2–3 weeks of incubation, cells were stained with 0.005% crystal violet and the number of colonies were counted. All experiments were performed in triplicates.

### Xenograft assay

NIH3T3 cells with EML4–ALK or EML4–ALK21S expression were subcutaneously transplanted into nude mice (5 × 10^6^ cells per mouse). Tumor volume was monitored every day and calculated by using formulation V = (L × W × W)/2. Mice were sacrificed and the tumors were harvested for further molecular and pathological analysis.

### Statistical analysis

Differences between groups were analyzed by One-way ANOVA or Two-way ANOVA and performed by Prism GraphPad software. *P* value < 0.05 was considered statistically significant. Error bars were represented with SEM.

## Supplementary information

Supplementary Information

Supplementary Movie S1

Supplementary Movie S2
