# Supplementary material for: Phase separation of EML4–ALK in firing downstream signaling and promoting lung tumorigenesis
Source: Cell Discov. 2021 May 11;7:33. doi: 10.1038/s41421-021-00270-5 (PMC8113584; doi:10.1038/s41421-021-00270-5)
Supplement: Supplementary file 1 — Supplementary Information [file 41421_2021_270_MOESM1_ESM.pdf]

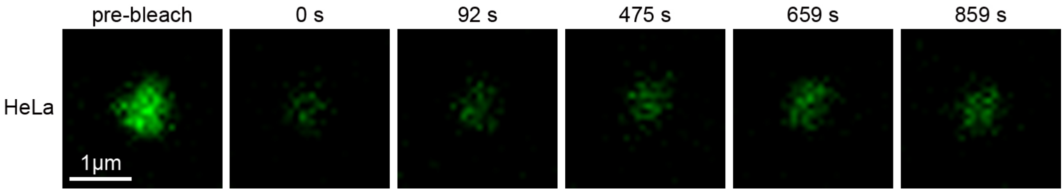

Supplementary Figure S1. FRAP images of GFP-EML4-ALK condensates with longer recovery time in HeLa cells.

Representative FRAP images of GFP-EML4-ALK condensates in HeLa cells. Time series of images were taken before and after photobleaching for up to 15 min. Scale bar, 1 μm.

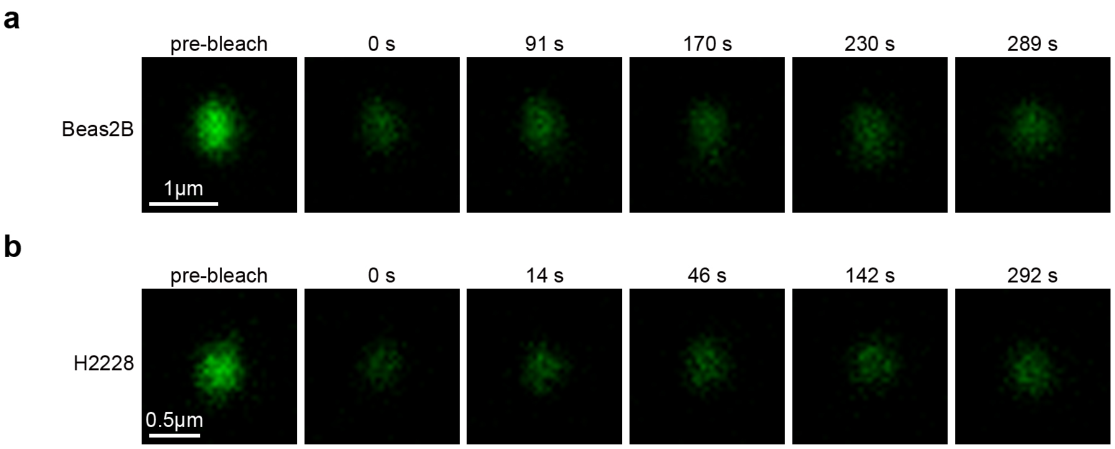

Supplementary Figure S2. FRAP images of GFP-EML4-ALK condensates in BEAS-2B and H2228 cells.

a Representative FRAP images of GFP-EML4-ALK condensates in BEAS-2B cells. The images were taken before and after photobleaching. Scale bar, 1 µm. **b** Representative FRAP images of GFP-EML4-ALK condensates in H2228 cells. The images were taken before and after photobleaching. Scale bar, 0.5 µm.

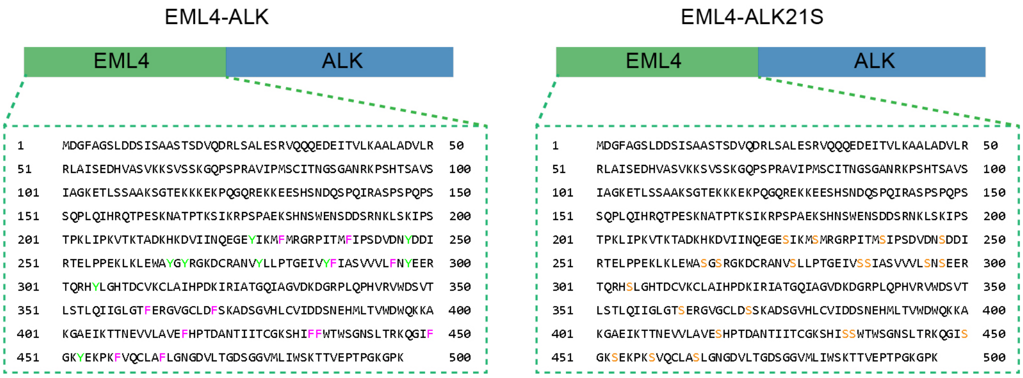

Supplementary Figure S3. Schematic diagram of wild-type EML4-ALK and EML4-ALK 21S mutant.

Tyrosine (indicated in green) and phenylalanine (indicated in magenta) were mutated to serine (indicated in orange).

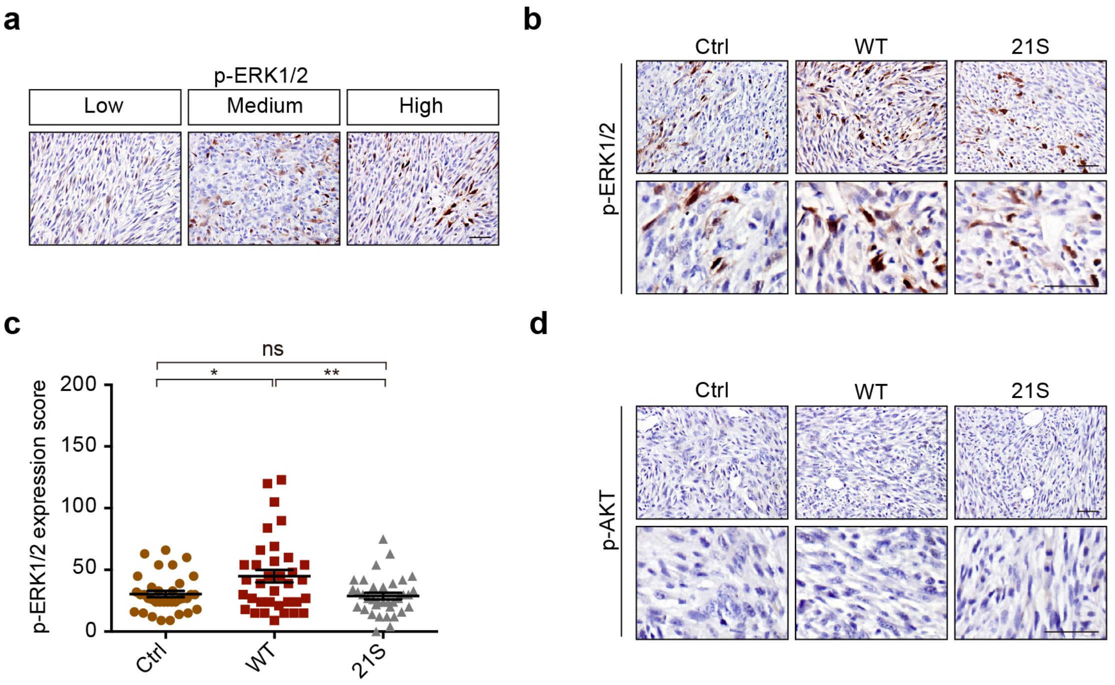

Supplementary Figure S4. The 21S mutations attenuate EML4-ALK-induced ERK1/2 hyperphosphorylation in tumors.

a Representative images of low, medium, high expression of p-ERK1/2. Scale bar, 50 μm. **b** Representative photos for p-ERK1/2 immunostaining in subcutaneous tumors derived from control, EML4-ALK and EML4-ALK21S group. Scale bar, 50 μm. **c** Statistical analysis of p-ERK1/2 immunostaining. **d** Representative photos for p-AKT immunostaining in subcutaneous tumors derived from control, EML4-ALK and EML4-ALK21S group. Scale bar, 50 μm. All data were shown as mean ± S.E.M. *$P<0.05$; **$P < 0.01$; ns, not significant. Ctrl, control; WT, EML4-ALK; 21S, EML4-ALK21S.

Supplementary Movie S1. GFP-EML4-ALK condensates undergo fusion

HeLa cells were transfected with GFP-EML4-ALK for 12 hours and GFP fluorescence was monitored through live imaging. Scale bar, 1 μm.

Supplementary Movie S2. GFP-EML4-N condensates undergo fusion

HeLa cells were transfected with GFP-EML4-N for 12 hours and GFP fluorescence was monitored through live imaging. Scale bar, 1 μm.
